# Human gut resistome can be country-specific

**DOI:** 10.7717/peerj.6389

**Published:** 2019-03-21

**Authors:** Yao Xia, Yanshan Zhu, Qier Li, Jiahai Lu

**Affiliations:** 1 Kunming Institute of Zoology, Chinese Academy of Sciences, Kunming, China; 2 Kunming College of Life Science, University of Chinese Academy of Sciences, Kunming, China; 3 School of Public Health, Sun Yat-sen University, Guangzhou, China; 4 School of Mathematics, Sun Yat-sen University, Guangzhou, China; 5 One Health Center of Excellence for Research & Training, Sun Yat-Sen University, Guangzhou, China; 6 Key Laboratory for Tropical Disease Control of Ministry of Education, Sun Yat-Sen University, Guangzhou, China

**Keywords:** Resistome, Antibiotic resistance gene, Metagenomics, Machine learning

## Abstract

The emergence and spread of antibiotic resistance have become emerging threats to human health. The human gut is a large reservoir for antibiotic resistance genes. The gut resistome may be influenced by many factors, but the consumption of antibiotics at both individual and country level should be one of the most significant factors. Previous studies have suggested that the gut resistome of different populations may vary, but lack quantitative characterization supported with relatively large datasets. In this study, we filled the gap by analyzing a large gut resistome dataset of 1,267 human gut samples of America, China, Denmark, and Spain. We built a stacking machine-learning model to determine whether the gut resistome can act as the sole feature to identify the nationality of an individual reliably. It turned out that the machine learning method could successfully identify American, Chinese, Danish, and Spanish populations with F1 score of 0.964, 0.987, 0.971, and 0.986, respectively. Our finding does highlight the significant differences in the composition of the gut resistome among different nationalities. Our study should be valuable for policy-makers to look into the influences of country-specific factors of the human gut resistome.

## Introduction

Recently, the emergence and spread of antibiotic resistance in bacteria have become global threats to human health (Ashbolt et al., 2013; Singer et al., 2016; Li et al., 2017; Goulas et al., 2018). For instance, antibiotic resistance may cause infections (e.g., pneumonia, tuberculosis) become harder to cure by the *loss of effectiveness* of currently available antibiotics. Typically, antibiotic resistance arises from antibiotic resistance genes (ARGs) carried by bacteria, which develops phenotypic resistance via a variety of mechanisms, including hydrolyzing the antibiotics directly, changing the antibiotic target and lowering concentrations of antibiotic through efflux pumps, etc. (Stokes & Gillings, 2011). ARGs exist in a wide range of environments, such as human, animal, soil, water, etc. (Zhang, Zhang & Fang, 2009; Ma et al., 2016; Hu, Gao & Zhu, 2017; Zhao et al., 2018). Antibiotics are very ancient, as are ARGs, and they have existed before humans started to recognize and utilize them (D’Costa et al., 2011; Bhullar et al., 2012; Segawa et al., 2013). In recent decades, however, the long-term abuse and misuse of antibiotics have significantly fostered and aggravated the spread of resistance.

Just like the microbiota, the total ARGs of an environmental sample can be referred to as the resistome. Previous studies have demonstrated that human gut is a large reservoir of ARGs (Sommer, Dantas & Church, 2009; Van Schaik, 2015). With the aim of understanding the overview of the human gut resistome, Hu et al. (2013) investigated 162 samples of human gut from three countries in 2013. They identified a total of 1,093 ARGs in all samples and found that even in the sample of the individual who has the least ARGs, there were still 33 ARGs types, indicating that human gut may have a strong antibiotic resistance potential. Yarygin et al. (2017) then created an electronic map to display the human gut resistome of different countries, which included 1,683 metagenomes data covering 15 countries. Recently, Feng et al. (2018) revealed the gut resistome of 180 healthy individuals from 11 different countries. It has been proposed that the resistome is an innate feature of the human gut microbiota, while other factors (such as extensive antibiotic usage) promote the acquisition, evolution, and spread of the resistome (Rampelli et al., 2015). In human gut microbiota, ARGs can exchange among different individual bacteria via horizontal transfer, and such exchanges may also occur during the immigration, even if the external bacteria are just passing through the intestine, which increases the risk that ARG-carrying pathogens will emerge. The human gut is a very complex environment and a variety of factors in addition to antibiotic usage (e.g., eating habits, medical history, and genetic factors) can make a difference on its resistome (Van Schaik, 2015; Hu et al., 2013). At the population level, some macro factors can also make an indirect impact on gut resistome (De Angelis et al., 2017). For instance, the residue level of antibiotics in food and drinking water may be different in different countries due to variation in policy and supervision of antibiotic use (Chen et al., 2017; Thorsten et al., 2010), as well as diverse personal eating preferences and medical situations, causing distinct long-term or short-term intake of antibiotics for individuals, which results in obvious alterations to the resistome.

At the population level, previous studies have demonstrated that the compositions of the gut resistome of people from different countries are significantly diverse. Forslund et al. (2013) compared country-level antibiotic by using data in parallel with studying the gut resistome in 252 samples of three countries, and observed that the differences in statistics of antibiotic use data match the resistance potential differences, indicating the antibiotic use, which is controlled by both individual-level living habitats and country-level policy, may be a major reason to influence the human gut resistome. Hu et al.’s (2013) also revealed that both types and abundances of ARGs in different populations of different countries are different. Feng et al. (2018) used a NMDS analysis to evaluate the similarity of resistome composition of 180 gut samples, and they found, in general, sample points from the same country have closer distance, although exceptions exist, which indicated that people from the same country may have more similar composition.

In previous studies, the sample size of the dataset used for investigating the resistome was not very large, as the metagenomic sequencing and its bioinformatics analysis are costly. In 2014, Li et al. (2014) published the integrated gene catalog (IGC) comprising 9,879,896 genes and 1,267 samples from four countries, Denmark, Spain, China, and America. Firstly, Li et al. (2014) constructed Illumina libraries for 249 new samples from the MetaHIT project with insert size of 350 bp, followed by high-throughput sequencing to obtain paired-end (PE) reads with length of 90 bp for each end. Then they combine the newly sequenced dataset with other three public datasets: (i) 139 gut samples the Human Microbiome Project (HMP) (Methé et al., 2012); (ii) 368 Chinese fecal samples (Qin et al., 2012); (iii) 511 European fecal samples from the MetaHIT project (Qin et al., 2010; Le Chatelier et al., 2013; Nielsen et al., 2014). The Illumina libraries of the samples from public datasets (ii) and (iii) were constructed and sequenced at BGI using the same protocol as the 249 new samples from the MetaHIT project, and the samples from public dataset (i) were processed by HMP sequencing centers using a similar protocol and platform (Illumina platform with 101 bp PE reads) (Li et al., 2014). Finally, they combined the newly sequenced dataset with the three public datasets and processed the raw reads using MOCAT (Kultima et al., 2012) for generating the IGC, where the nationality of each sample was recorded (Li et al., 2014). As for the gene profile, they mapped the high quality reads from each sample to the gene cataloge using the criterion of identity ≥95% (Li et al., 2014) and then computed the relative abundance of each gene using the method from a previous study performed by Qin et al. (2012).

Although, the microbiome composition and the country-specific differences in overall microbiome composition in IGC have been showed in Li et al.’s (2014) study, the resistome in IGC has not been studied yet. Therefore, in this study, we have two particular concerns: (i) revealing the human gut resistome using the IGC; (ii) investigating the similarity of the composition of gut resistome among populations in different countries. We first compared the total resistance potential for four countries and identified ARG types and subtypes that significantly differ. Then, we used the gut resistome solely as input features to build a stacking machine-learning model and evaluate whether it could recognize the correct nationality of a certain individual.

## Material and methods

### Dataset description

The IGC contains 1,267 samples from four countries, Denmark (*n* = 401), Spain (*n* = 359), China (*n* = 368), America (*n* = 139). Reads for gut samples were independently processed (quality control, removal of human sequences, assembling, assembly revision, and gene prediction) using MOCAT for generating a non-redundant gene cataloge (Li et al., 2014). Then the high quality reads from each sample were aligned against the gene cataloge using the criterion of identity ≥95% (Li et al., 2014) and the relative abundance of each gene in the cataloge was computed by the following steps (Qin et al., 2012):

(1) $${b_i} = \displaystyle{{{x_i}} \over {{L_i}}}$$

where *b*_*i*_ is copy number of the *i*th gene in the cataloge, *x*_*i*_ is time when gene *I* can be detected in the sample (the mapped reads of the *i*th gene) and *L*_*i*_ is the length of the *i*th gene.

(2) $${a_i} = \displaystyle{{{b_i}} \over {\sum\nolimits_j {{b_j}} }}$$

where *a*_*i*_ is the relative abundance of the *i*th gene, and Σ_*j*_*b*_*j*_ is the total copy number of all genes in the sample.

### Search for ARGs

The raw sequences (integrated non-redundant gene catalog, IGC, nucleotide sequences, fasta) of all genes in IGC were downloaded in http://meta.genomics.cn/meta/dataTools. All the gene sequences were searched for ARGs against the well-structured ARGminer database v0.1.6 (https://bench.cs.vt.edu/ftp/argminer/release/) using Diamond (Buchfink, Xie & Huson, 2015) blastx model with *E*-value < 1*e−*10. A gene sequence was annotated as ARG if its best hit has the similarity higher than 80% and coverage higher than 70% (Hu et al., 2013). Identified ARGs were assigned into types (e.g., tetracycline, multidrug, bacitracin) and subtypes (e.g., tetA, MdtL, uppP).

### Abundance and diversity of ARGs

The raw profile data (gene abundance profile table for 1,267 samples) of IGC dataset was downloaded in http://meta.genomics.cn/meta/dataTools. The relative abundance of a certain ARG type or subtype was calculated as the sum of the relative abundance of genes annotated to the same type or subtype. A Kruskal–Wallis test was adopted to compare the difference of relative abundance of every ARG type and subtype among countries. In this step, as our aim is to identify ARG types and subtypes that have significant difference among countries, therefore we did not perform further pair-wise tests.

For each sample, the total relative abundance of ARG subtypes was calculated. The Kruskal–Wallis test was used for comparing the difference of ARG diversity among four countries and Wilcox pair-wise test was used to compare the difference between every two countries, where the *p*-value was adjusted using Bonferroni method.

### Country-specific classification based on resistome

As the relative abundances of ARG subtypes are very close to 0, we firstly performed a log transformation using the following equation:

(3) $${L_i} = 10 + {\log _{10}}({a_i} + 1E - 10)$$

where *L*_*i*_ and *a*_*i*_ are the log transformation and relative abundance of the *i*th ARG subtype, respectively, and 1*E−*10 is a scientific notation.

For building the machine learning model, we used the 1D vector containing log transformations of profiles of all ARG subtypes in a sample as its input feature, and nationality of the sample as its output label. The 1,267 samples were stratified and divided randomly into 70% (886) training set and 30% (381) testing set. The training set was used for models training, tuning, and validating, and the testing set was used for the final model evaluation.

We selected six diverse-type of machine learning models (i.e., Logistic Regression, Support Vector, K-Nearest Neighbor, Random Forest, Gradient Boosting Decision Tree, and Multi-layer Perception) as the basic models. For each model, the hyper-parameters were tuned based on the training set via GridSearchCV. The cross-validation strategy was set to stratifying and randomly splitting the training set into 70% sub-training set and 30% sub-testing set for cross-validation, and accuracy was calculated. This process repeated 10 times and the mean of accuracy is used to select the best parameters combination.

Then six basic models with best parameters were used for stacking. For each basic model, a fivefold cross-validation process was performed and the predictions from the process were stored for training the meta-classifier, a Logistic Regression Classifier. In brief, the dataset is split into fivefolds, and in five successive rounds, fourfolds are used to fit each basic model, and then it is applied to the remaining onefold for making predictions, which are then stacked and provided to the meta-classifier. GirdSearchCV was used again for tuning the parameters of the meta-classifier of the stacking model using the same cross-validation strategy as the indicator as described before. The best parameter combination that maximized the mean accuracy of 10 repetitions was set, and then the stacking model was fitted with the whole training set.

Finally, the fitted stacking model was applied to the test set for predicting the nationality of each individual. The precision, recall and F1-score for each prediction were calculated for model evaluation. The machine learning modeling was conducted using *sklearn* and *mlxtend* package in a python environment.

## Results

### Human gut resistome vary significantly among countries

**Figure 1:**
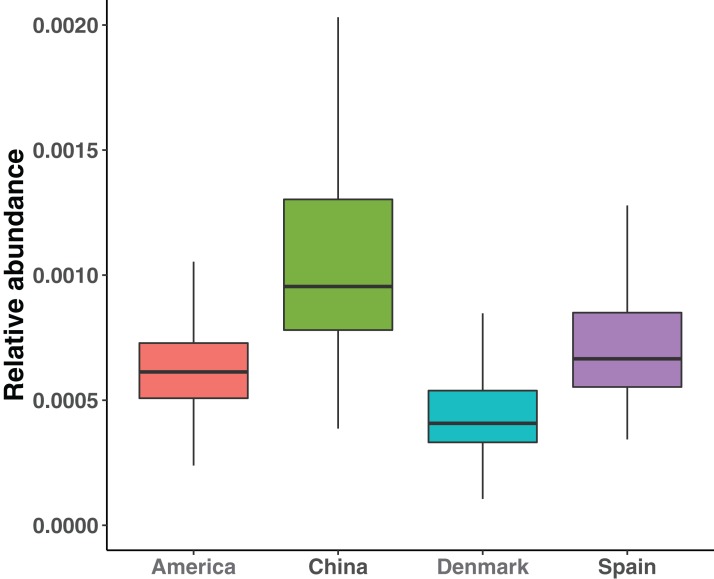
Comparison of the mean of abundance of total ARG subtypes of samples among four countries. The boxes with different colors denote the relative abundance of total ARG subtypes of samples from different countries, where red box denotes America, green box denotes China, blue box denotes Denmark and purple box denotes Spain.

In total, 9,879,896 genes were queried against the ARGminer database and 2,547 (0.26%) genes were annotated as ARGs. According to ARGminer, 2,547 ARG-like genes were grouped into 332 subtypes and 19 types. We compared the resistance potential, which was defined as the mean of total relative abundance ARG subtypes of all samples from the same country, among populations from four countries (Fig. 1). We found that Chinese harbored the highest resistance potential and Danish population harbored the least abundant ARGs. The Kruskal–Wallis test shows that there are significant differences in four countries (*p*-value < 0.01), and with Wilcox pair-wise test, all pairs show significant difference (adjusted *p*-value < 0.01).

**Figure 2:**
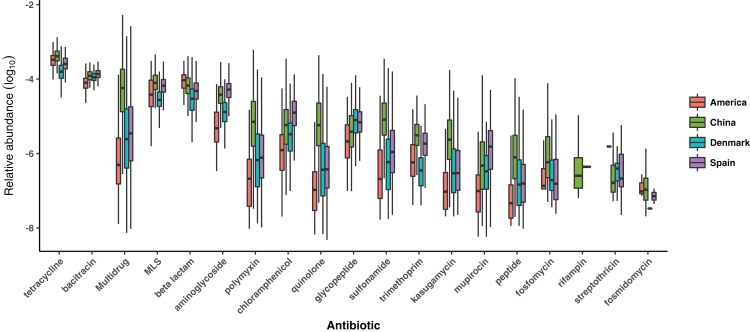
Comparison of the ARG types among populations from four countries. The boxes with different colors denote the log-transformed relative abundance of ARG types of samples from different countries, where red boxes denote America, green boxes denote China, blue boxes denote Denmark and purple boxes denote Spain.

For each of the 19 ARG types, we compared its relative abundance among four countries (Fig. 2). Teracyline, Bacitracin, Multidrug, Macrolide–Lincosamide–Streptogramin and Beta lactam are the most abundant ARG types in human gut resistome. All ARG types were found significantly different among four countries via a Kruskal–Wallis (*p*-values < 0.05). In most cases, populations from China and Spain showed significantly higher relative abundance of ARG type, comparing with other two countries.

**Figure 3:**
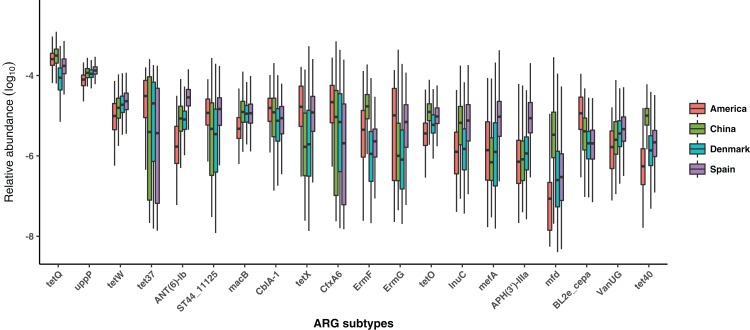
Comparison of the most abundant 20 ARG subtypes among populations from four countries. The boxes with different colors denote the log-transformed relative abundance of the most abundant 20 ARG subtypes of samples from different countries, where red boxes denote America, green boxes denote China, blue boxes denote Denmark and purple boxes denote Spain.

For each of the 332 ARG subtypes, we also compared its relative abundance among four countries. The 20 most abundant subtypes in all samples were displayed in Fig. 3. TetQ (Tetracyline), uppP (Bacitracin), tetW (Tetracyline), tet37 (Tetracyline), and ANT(6)—lb (Aminoglycoside) were the five most abundant subtypes. Through a Kruskal–Wallis test, 301/332 (90.66%) ARG subtypes were found significantly different (*p-*value < 0.05) among populations from four countries.

### Country-specific classification based on resistome

The validation results were listed in Table 1 and displayed using the macro mean for precision, recall and F1 score from 10 times random testing. The Logistic Regression performed best among all and K-Neighbors performed worst. The low standard deviation indicated that the models were stable for 10 times repetitions. The evaluation was conducted using the stacking model on the testing set. The precisions, recalls and F1 scores for identifying four countries were listed in Table 2. To identify Spanish population, the stacking model had the highest precision 0.991, and to identify Chinese population, the stacking model has the highest recall 0.991 and the highest F1 score of 0.987. The mean values of precision, recall and F1 score for the stacking model were 0.979, 0.979, and 0.979, respectively.

**Table 1:** The results of validations of six basic models.

Models	Precision	Precision SD	Recall	Recall SD	F1 score	F1 score SD
LogisticRegressionCV	0.962	0.013	0.959	0.011	0.960	0.012
XGBClassifier	0.959	0.012	0.958	0.014	0.959	0.013
MLPClassifier	0.954	0.011	0.954	0.013	0.954	0.012
SVC	0.942	0.014	0.935	0.017	0.938	0.015
RandomForestClassifier	0.940	0.014	0.922	0.023	0.929	0.020
KNeighborsClassifier	0.881	0.019	0.866	0.024	0.871	0.022

**Table 2:** Stacking model evaluation.

Country	Precision	Recall	F1 score	Sample number
America	0.976	0.952	0.964	42
China	0.982	0.991	0.987	111
Denmark	0.967	0.975	0.971	120
Spain	0.991	0.981	0.986	108
Mean/Sum	0.979	0.979	0.979	381

## Discussion

The IGC has been widely used as a reference gene catalog for many metagenomics studies, but the resistome in IGC dataset has not been investigated yet. Our results filled this gap and provided a border overview of the human gut resistome. We found that Chinese population harbored the most abundant and the most diverse ARGs in gut microbiota, where the antibiotic overuse may be the major reason. In China, around 75% of patients with seasonal influenza are estimated to be prescribed antibiotics, which may not be necessary in most cases (Heddini et al., 2009). In addition, Forslund et al. (2013) revealed that antibiotic used in animals are also contributing to human gut resistome, which is consistent with expectations from previous research into a ‘‘farm-to-fork’’ connection. One possible way for the link may be that the antibiotic residue in meat increases the intake of antibiotic potentially. Several studies have found that the exchange of ARGs between bacteria from farm animals, human food and human-associated bacteria (clinical isolates, and so on) by horizontal gene transfer, explaining that some ARGs in human gut microbiota may come from animal source (Teale, 2002; Sørum & L’Abée-Lund, 2002; Smillie et al., 2011). This may be another reason why Chinese harbored very abundant and diverse resistome. Although there are no official data on antibiotic use in Chinese agriculture, Krishnasamy, Otte & Silbergeld (2015) estimated that 38.5 million kg antibiotic were used in 2012 in China’s production of swine and poultry, which is an alarming volume.

It should be noted that antibiotic consumption is just one reason partly explaining the difference among countries. The force shaping and factors influencing gut resistome are far from known, especially at individual level. A plenty of factors, such as eating habitats, lifestyle, genetic background, living environment, bacterial infections, etc. could contribute to, in varying degree, the emergence and spread of ARGs in gut microbiota. The antibiotic may serve as a selector, and eliminate the bacteria without ARG, making a preferable environmental for bacteria with ARG. In addition, some of antibiotic resistance mechanisms are not linked to antibiotic resistance originally, such as efflux pumps, which is reported to be involved in signal trafficking or resistance to toxic compounds at the first time, indicating that the bacteria themselves are also playing an important role in affecting resistome. In addition, ARGs can transfer from each other within microbiota, which makes the resistome more complex for understanding as the ecological factors of gut microbiota also play an important role. Some studies report that natural environments, such as air, water, soil, etc. also contain ARGs, which make it possible for human to get ARGs through breath, drinking, touch.

The possible factors influencing gut resistome described above could have evident difference among countries. Therefore, it is reasonable to deduct that human gut resistome could vary significantly across country-specific populations. We wonder whether such difference is significant enough so that resistome could be used as a predictor for classifying individual into different country. In this study, we used a stacking machine learning model to test this hypothesis. Our results demonstrated high precision, recall and F1 score for identifying populations from all four countries, suggesting that the gut resistome could serve as an effective predictor.

Although to compare the difference of resistome between populations has been conducted in previous studies, our study provided a new idea to display such difference. We proposed that the resistome could be used as features to identify the nationalities of individuals correctively using machine learning only when the compositions of gut resistome of populations in different countries were obviously various. This may be more intuitive than traditional methods for studying the similarity of composition, such as PCA, especially when the sample size is very large, which will make the PCA plot hard to read. This idea could be also extended to compare the difference of overall microbiome among different treatments, or to find and evaluate markers to identify the correct treatment that a certain sample belongs to.

One important question that we should consider: “Does the predicted gene profile reflect the underlying true gene profile in the metagenome samples?” In metagenomics studies, millions genes with different length would be predicted. To represent the true abundance of each gene, the gene profiling method must be considered and chosen carefully. For instance, in a resistome study performed by Munk et al. (2018), FPKM was used to represent the gene profile. In the IGC, the relative abundance of each gene was computed using the method from Qin et al.’s (2012) study, the accuracy of which was evaluated using the method developed by Audic & Claverie (1997). The result showed that there is a very low detection error rate (Qin et al., 2012), suggesting that the method for gene profiling in the IGC is reliable. Therefore in our study, we performed our analyses using the gene profile of the IGC directly.

There are many ARG databases and related tools, such as ARDB (Liu & Pop, 2009), CARD (McArthur et al., 2013), ARG-ANNOT (Gupta et al., 2014), DeepARG-DB (Arango-Argoty et al., 2018), pairwise comparative modeling (Ruppe et al., 2017), etc. The methods for generating the ARG database and their updating varied obviously. To use any of them solely may have a risk that some genes will not be annotated as ARGs due to the possible incompleteness of the database. Therefore we chose ARGminer (Arango-Argoty et al., 2018), a recently developed database that is curated via a crowd-sourcing platform, to annotate the IGC. The ARGminer integrated ARGs from CARD, ARDB, DeepARG-DB, UniProt (The UniProt Consortium, 2015), and the MEGARes (Lakin et al., 2017) that incorporates genes from the ARG-ANNOT, RESFINDER (Zankari et al., 2012), and the Lahey Clinic beta-lactamase archive (Bush & Jacoby, 2010). After collecting, the ARGs in ARGminer were curated using a crowd sourcing method via a web platform. The crowd sourcing method has been validated, and the results showed that crowdsourcing workers are as accurate as experts in curating ARGs (Arango-Argoty et al., 2018). Although ARGminer provides a novel and effective way to integrate different ARG databases and curate ARGs, we still cannot conclude that the annotation via ARGminer is the most precise, as crowdsourcing workers cannot promise 100% accuracy in curating ARGs. Some potential bias may exist due to the crowd sourcing method. This is the main limitation of our study.

## Conclusions

Although in real life, it is not necessary to use gut resistome to identify a person’s nationality in practice, our results, in an indirect way, revealed that the differences of gut resistome among populations of different countries are very significant. These findings should be valuable for policy-makers to look into the influences of country-specific factors on the human gut resistome. However, country-specific population diversity is too high, which makes the essential reason why the country-specific difference in gut resistome difficult to explain precisely. Further studies could make efforts to compare populations with more internally consistent characteristics, such as populations from different cities in the same country.

## Additional Information and Declarations

### Data Availability

The following information was supplied regarding data availability: The raw sequences (Integrated non-redundant gene catalog, IGC, nucleotide sequences, fasta) of all genes in IGC and the raw profile data (Gene abundance profile table for 1,267 samples) of IGC dataset were downloaded from http://meta.genomics.cn/meta/dataTools.
